# Ionic Mechanisms of Disopyramide Prolonging Action Potential Duration in Human-Induced Pluripotent Stem Cell-Derived Cardiomyocytes From a Patient With Short QT Syndrome Type 1

**DOI:** 10.3389/fphar.2020.554422

**Published:** 2020-10-12

**Authors:** Huan Lan, Qiang Xu, Ibrahim El-Battrawy, Rujia Zhong, Xin Li, Siegfried Lang, Lukas Cyganek, Martin Borggrefe, Xiaobo Zhou, Ibrahim Akin

**Affiliations:** ^1^Key Laboratory of Medical Electrophysiology of Ministry of Education and Medical Electrophysiological Key Laboratory of Sichuan Province, Institute of Cardiovascular Research, Southwest Medical University, Luzhou, China; ^2^First Department of Medicine, Faculty of Medicine, University Medical Centre Mannheim (UMM), University of Heidelberg, Mannheim, Germany; ^3^Department of Histology and Embryology, Southwest Medical University, Luzhou, China; ^4^DZHK (German Center for Cardiovascular Research), Partner Site Heidelberg-Mannheim, Mannheim, Germany; ^5^DZHK (German Center for Cardiovascular Research), Partner Site Göttingen, Göttingen, Germany; ^6^Stem Cell Unit, Clinic for Cardiology and Pneumology, University Medical Center Göttingen, Göttingen, Germany

**Keywords:** short QT syndrome, arrhythmias, antiarrhythmic drugs, disopyramide, human-induced pluripotent stem cell-derived cardiomyocytes

## Abstract

Short QT syndrome (SQTS) is associated with tachyarrhythmias and sudden cardiac death. So far, only quinidine has been demonstrated to be effective in patients with SQTS type 1(SQTS1). The aim of this study was to investigate the mechanisms of disopyramide underlying its antiarrhythmic effects in SQTS1 with the N588K mutation in HERG channel. Human-induced pluripotent stem cell–derived cardiomyocytes (hiPSC-CMs) from a patient with SQTS1 and a healthy donor, patch clamp, and calcium imaging measurements were employed to assess the drug effects. Disopyramide prolonged the action potential duration (APD) in hiPSC-CMs from a SQTS1-patient (SQTS1-hiPSC-CMs). In spontaneously beating SQTS1-hiPSC-CMs challenged by carbachol plus epinephrine, disopyramide reduced the arrhythmic events. Disopyramide enhanced the inward L-type calcium channel current (I_Ca-L_), the late sodium channel current (late I_Na_) and the Na/Ca exchanger current (I_NCX_), but it reduced the outward small-conductance calcium-activated potassium channel current (I_SK_), leading to APD-prolongation. Disopyramide displayed no effects on the rapidly and slowly activating delayed rectifier and ATP-sensitive potassium channel currents. In hiPSC-CMs from the healthy donor, disopyramide reduced peak I_Na_, I_Ca-L_, I_Kr_, and I_SK_ but enhanced late I_Na_ and I_NCX_. The results demonstrated that disopyramide may be effective for preventing tachyarrhythmias in SQTS1-patients carrying the N588K mutation in HERG channel by APD-prolongation *via* enhancing I_Ca-L_, late I_Na_, I_NCX_, and reducing I_SK_.

## Introduction

Short QT syndrome (SQTS) is a rare, inheritable cardiac channelopathy associated with abbreviated corrected QT interval (QTc), tachyarrhythmias and sudden cardiac death (SCD) (Gussak et al., 2000; Brugada et al., 2004). So far, worldwide more than 200 SQTS-patients with different gene mutations have been reported and different types of SQTS have been described (Bjerregaard, 2018; Campuzano et al., 2018). SQTS types 1–3 are linked to a gain of function of potassium channels led by mutations in the KCNH2 (SQTS1), KCNQ1 (SQTS2), and KCNJ2 (SQTS3) gene. SQTS types 4-6 are linked to a loss of function of calcium channels resulting from mutations in CACNA1C (SQTS4), CACNB2 (SQTS5), and CACNA2D1 (SQTS6) gene. Recently, a mutation in the cardiac Cl/HCO3 exchanger AE3 was detected in two SQTS-families (Thorsen et al., 2017; Campuzano et al., 2018).

The diagnostic and treatment approaches are still challenging because the prevalence of the disease is very low. Although implantable cardioverter defibrillator (ICD) can be useful for terminating arrhythmias in SQTS-patients, ICD cannot be used for every patient (Giustetto et al., 2006; Mazzanti et al., 2014). Therefore, pharmacotherapy is required at least for some SQTS-patients.

To date, only a limited number of drugs have been examined in a small number of patients with SQTS1 (Abriel and Rougier, 2013), among which only one drug quinidine has been shown to be effective in the treatment (Mizobuchi et al., 2008; Giustetto et al., 2011). Quinidine cannot be used in some patients due to its severe side effects. Hence, searching for further drugs is urgently required. SQTS1 is caused by a gain-of-function of the KCNH2 channel. KCNH2 channel blockers should prolong QT interval and hence suppress arrhythmias in SQTS1patients. Surprisingly, clinical data demonstrated that some KCNH2 channel blockers including sotalol and ibutilide failed to prolong QT-interval in SQTS1-patients with KCNH2 mutations (Gaita et al., 2004). The reason for ineffectiveness of drugs is that the mutation (N588K) in the KCNH2 channel impairs inactivation of the channel and renders the channel resistant to blockers, which have the highest affinity to the inactivated state of KCNH2 channels. Quinidine has high affinity to both the open and inactivated states of KCNH2 channels. Therefore, its affinity is only partially reduced and it is effective in treating SQTS1. Given that a mutation in KCNH2 (or other channels) may change the sensitivity of channels to drugs (McPate et al., 2008), other factors like epigenetic and environmental factors may also influence drug effects. In different types of cells or in the same type of cells under different states, a drug may show different effects. It will be important to test drug effects in “diseased” cells if we want to know the efficacy of a drug for treating the disease.

Disopyramide is a multiple channel blocker, mainly a sodium channel blocker (Morady et al., 1982; Sunami et al., 1991; Koumi et al., 1992). It has been used for atrial and ventricular arrhythmias (Morady et al., 1982; Verlinden and Coons, 2015). It was also tested in a SQTS-patient with unknown genotype (Mizobuchi et al., 2008). Although it normalized QT interval in that patient, its efficacy for treating SQTS is unclear because only one patient was recruited for the study. Disopyramide effects on HERG (also called I_Kr_ or KCNH2) channels, either the wild type or mutated, were investigated in different types of cells (Virag et al., 1998; McPate et al., 2006; El Harchi et al., 2012a; El Harchi et al., 2012b), but not in SQTS-cardiomyocytes. Recently, disopyramide was tested in hiPSC-CMs from a patient with SQTS1 and beneficial effects (APD-prolonging and antiarrhythmic effects) were detected (Shinnawi et al., 2019). However, in that study, the mechanisms of disopyramide underlying the APD-prolonging and antiarrhythmic effects were not investigated.

This study was designed to investigate the ionic mechanisms for the APD-prolonging and antiarrhythmic effects of disopyramide using the cellular model of SQTS1-hiPSC-CMs established recently by our group.

## Methods

### Ethics Statement

A skin biopsy from a SQTS1 patient was obtained with written informed consent. The study was approved by the Ethics Committee of the Medical Faculty Mannheim, University of Heidelberg (approval numbers: 2018-565N-MA), the Ethics Committee of University Medical Center Göttingen (approval number: 10/9/15), and the Ethics Committee of Southwest Medical University (approval number:KY2013019). The study was carried out in accordance with the approved guidelines and conducted in accordance with the Helsinki Declaration of 1975 (https://www.wma.net/what-we-do/medical-ethics/declaration-of-helsinki/), revised in 2013.

### Clinical Data

The fibroblasts for iPS cell generation were from a 29-year-old male patient with familial SQTS1 carrying a missense mutation (C to G substitution at nucleotide 1764). This mutation results in substitution of an amino acid at the position of 588 from asparagine to lysine (N588K) in the KCNH2 (also called HERG or *I_Kr_*) channel. The clinical data of the patient has been provided in our recent publication (El-Battrawy et al., 2018).

### Generation of Human iPS Cells

The methods for the generation of iPS cells from the patient and a healthy donor have been described in our previous study (El-Battrawy et al., 2018). Briefly, human iPS cells (hiPSCs) were generated from primary human fibroblasts derived from a skin biopsy. The hiPSC line was generated under feeder free culture conditions using the integration-free CytoTune-iPS 2.0 Sendai Reprogramming Kit (Thermo Fisher Scientific, #A16517). The Kit contains the reprogramming factors OCT4, KLF4, SOX2, c-MYC and was used according to manufacturer’s instructions with modifications. The generated hiPSCs were characterized for their pluripotency and their *in vitro* differentiation potential (El-Battrawy et al., 2018).

### Generation of hiPSC-CMs

The hiPSCs were cultured without feeder cells and differentiated into hiPSC-CMs as described with some modifications (Tiburcy et al., 2017). In our lab the differentiation of hiPS cells into cardiomyocytes (hiPSC-CMs) is regularly performed every 2 to 3 weeks. The hiPSC-CMs from different differentiations were used for studies and the data were combined. Three clones of the hiPSCs were alternately used for the differentiation. At 40 to 60 days of culture with basic culture medium, cardiomyocytes were dissociated from 24 well plates and plated on Matrigel-coated 3.5 cm petri dishes for patch-clamp and calcium transient measurements.

### Patch-Clamp

Standard patch-clamp recording techniques were used to measure the action potential (AP) and channel currents in the whole-cell configuration at room temperature. Patch electrodes were pulled from borosilicate glass capillaries (MTW 150F; world Precision Instruments, Inc., Sarasota, FL) using a DMZ-Universal Puller (Zeitz-Instrumente Vertriebs GmbH, Martinsried, Germany) and filled with pre-filtered pipette solution (see below). Pipette resistance ranged from 1–2 MΩ and 4–5 MΩ for current and AP measurements, respectively. Electrode offset potentials were zero-adjusted before a Giga-seal was formed. After a Giga-seal was obtained, fast capacitance was first compensated and then the membrane under the pipette tip was disrupted by negative pressure to establish the whole-cell configuration. Signals were acquired at 10 kHz and filtered at 2 kHz with the Axon 200B amplifier and Digidata 1440A digitizer hardware as well as pClamp10.2 software (Molecular Devices, Sunnyvale, CA). APs were recorded in current clamp mode. For recording APs, brief current pulses (2 ms, 1 nA) were applied with different frequencies to trigger APs.

The bath solution (PSS) for AP measurements contained (mmol/l): 130 NaCl, 5.9 KCl, 2.4 CaCl_2_, 1.2 MgCl_2_, 11 glucose, 10 HEPES, pH 7.4 (NaOH). The pipette solution contained (mmol/l): 10 HEPES, 126 KCl, 6 NaCl, 1.2 MgCl_2_, 5 EGTA, 11 glucose and 1 MgATP, pH 7.2 (KOH).

The bath solution for L-type (I_Ca-L_) calcium channel current recordings contained (mmol/l): 140 TEA-Cl, 5 CaCl_2_, 1 MgCl_2_, 0.01 E-4031, 10 HEPES, 0.02 TTX, 3 4-AP, pH 7.4 (CsOH). Microelectrodes were filled with (mmol/l): 6 NaCl, 135 CsCl, 2 CaCl_2_, 3 MgATP, 2 TEA-Cl, 5 EGTA, 10 HEPES, pH7.2 (CsOH).

The bath solution for Na^+^-Ca^2+^ exchanger current (I_NCX_) measurements contained (mmol/l): 135 NaCl, 10 CsCl, 2 CaCl_2_, 1 MgCl_2_, 10 Hepes, 10 glucose, 0.01 nifedipine, 0.1 niflumic acid, 0.05 lidocaine, 0.02 dihydroouabain, pH 7.4 (CsOH). Microelectrodes were filled with (mmol/l): 10 NaOH, 150 CsOH, 2 CaCl_2_, 1 MgCl_2_, 75 aspartic acid, 5 EGTA, pH7.2 (CsOH). NiCl_2_ (5mM) was used to separate I_NCX_ from other currents. I_NCX_ was defined as NiCl_2_-sensitive current.

The bath solution for Na+ current measurements contained (mmol/l): 135 NaCl, 20 CsCl, 1.8 CaCl_2_, 1 MgCl_2_, 10 Hepes, 10 glucose, 0.001 nifedipine, pH 7.4 (CsOH). Microelectrodes were filled with (mmol/l): 10 NaCl, 135 CsCl, 2 CaCl_2_, 3 MgATP, 2 TEA-Cl, 5 EGTA, and 10 HEPES (pH7.2 CsOH).

The bath solution for K^+^ channel current measurements contained (mmol/l): 130 NaCl, 5.9 KCl, 2.4 CaCl_2_, 1.2 MgCl_2_, 11 glucose, 10 HEPES, pH 7.4 (NaOH). For slowly delayed rectifier (I_Ks_) measurements, 10 µM nifedipine, 3 mM 4-AP and 10 µM TTX were added. The pipette solution contains 10 mM HEPES, 126 mM KCl, 6 mM NaCl, 1.2 mM MgCl_2_, 5 mM EGTA, 11 mM glucose, and 1 mM MgATP, pH 7.4 (KOH). For measuring small conductance calcium-activated potassium channel currents (I_SK_), appropriate CaCl_2_ was added to get the free-Ca^2+^ concentration of 0.5 µM according to the calculation by the software MAXCHELATOR (http://web.stanford.edu/~cpatton/downloads.htm). For measuring ATP-sensitive K^+^ channel currents (I_KATP_), the ATP-free pipette solution was used. I_Ks_ was defined as 3R4S-chromanol 293B-sensitive, I_KATP_ as nicorandil-sensitive and I_SK_ as apamin-sensitive currents.

To separate I_Kr_ from other K^+^ channel currents, the Cs^+^ currents conducted by KCNH2 (*I_Kr_*) channels were measured. External solution for Cs^+^ currents (mmol/L): 140 CsCl, 2 MgCl_2_, 10 HEPES, 10 Glucose, pH=7.4 (CsOH). Pipette solution: 140 CsCl, 2 MgCl_2_, 10 HEPES, 10 EGTA, pH=7.2 (CsOH).

### Measurement of Intracellular Calcium Transients

To measure the intracellular Ca^2+^ transients, cells were loaded with the fluorescent Ca^2+^-indicator Fluo-3 AM. First, 1.5 ml PSS (see above) was added into a petri dish with hiPSC-CMs cultured for 2 to 4 days. Then, 50 µg of the membrane permeable acetoxymethyl ester derivative of Fluo-3 was dissolved in 44 µl of the Pluronic F-127 stock solution (20% w/v in DMSO) to get a 1 mM Fluo-3 AM stock solution, which can be stored at -20°C for a maximum of 1 week. Next, 15 µl of the Fluo-3 AM stock solution were added into 1.5 ml PSS resulting in a final concentration of 10 µM Fluo-3 and the dish was agitated carefully. The cells were incubated at room temperature for 10 min in an optically opaque box to protect from light. Thereafter, the PSS was carefully sucked out and discarded. The cells were washed with PSS for 4–5 times. Finally, the cells in PSS were kept at room temperature for about 30 min for de-esterification before measurements. After de-esterification, the fluorescence of the cells was measured by using Cairn Optoscan calcium imaging system (Cairn Research, UK). Fluorescence is excited by 488 nm and emitted at 520 nm. The calcium transients were recorded at room temperature.

### Drugs

Disopyramide is from SigmaAldrich. The drug was applied to a cell sequentially from low to high concentrations by a perfusion pipette. The tested concentrations were selected according to previous or our preliminary studies in hiPSC-CMs. E-4031, chromanol 293B, nifedipine, NiCl_2_, niflunic acid, lidocaine, and dihydroouabain are from Sigma Aldrich, 4-AP from RBI, apamin from Alomone Labs, TTX from Carl Roth. E-4031, NiCl_2_, TTX, 4-AP, apamin, niflumic acid, and dihydrooubain were dissolved in H_2_O. Nifedipine, and chromanol 293B were dissolved in DMSO, lidocaine in ethanol. Stock solutions were kept at -20 °C.

### Statistical Analysis

Data are shown as mean ± SEM and were analyzed using InStat^©^ (GraphPad, San Diego, USA) and SigmaPlot 11.0 (Systat GmbH, Germany). By analyzing the data with the Kolmogorov Smirnov test, it was decided whether parametric or non-parametric tests were used for analysis. For parametric data of more than two groups, multiple comparisons with one-way ANOVA and Holm-Sidak post-test were performed. For repeated measurements, the method of one-way repeated measures ANOVA with Holm-Sidak post-test was applied. The I-V curve data were analyzed with mixed model analysis using repeated values for the same cells measured as control and treatment at different voltages. Paired t-test was used for comparisons of data before and after application of a drug. p<0.05 (two-tailed) was considered significant.

## Results

### Disopyramide Prolonged the Action Potential Duration in SQTS1-hiPSC-CMs

**Figure 1:**
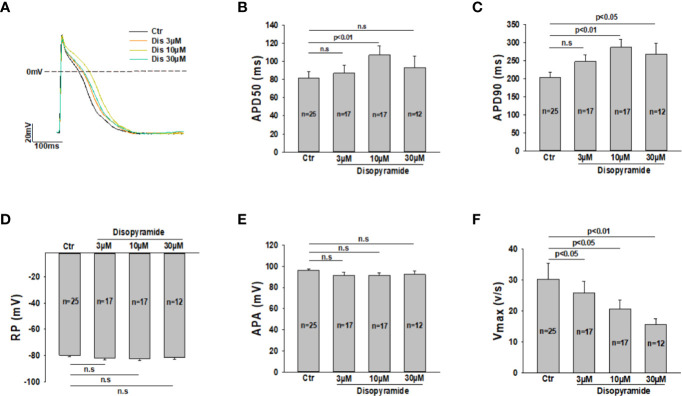
Effects of disopyramide on action potentials in SQTS1-hiPSC-CMs. Action potentials were recorded at 1 Hz. Disopyramide was applied to cells sequentially from low to high concentration (3 µM, 10 µM and 30 µM). **(A)** Representative action potential traces in absence (Ctr) and presence of 3 µM, 10 µM, and 30 µM disopyramide. **(B)** Averaged values of action potential duration at 50% repolarization (APD50). **(C)** Averaged values of action potential duration at 90% repolarization (APD90). **(D)** Averaged values of resting potential (RP). **(E)** Averaged values of action potential amplitude (APA). **(F)** Averaged values of maximal depolarization velocity (Vmax). Shown are mean ± SEM, n represents number of cells. The statistical significance was examined by One Way Repeated Measures ANOVA followed by Holm-Sidak method. ns, not significant.

Although disopyramide has been shown to prolong action potential duration and suppress arrhythmias in SQTS cells, we checked both effects in our SQTS1-hiPSC-CMs before investigating its ionic mechanisms. The AP parameters including action potential amplitude (APA), the maximal upstroke velocity (Vmax) and action potential durations at 50% and 90% repolarization (APD50 and APD90) were analyzed in the presence of disopyramide. Indeed, disopyramide at the concentration of 10 and 30 µM prolonged APD90, while at 10 µM significantly prolonged APD50 (**Figures 1A–C**). At all the tested concentrations, disopyramide did not significantly affect RP, APA (**Figures 1D, E**), but reduced Vmax in a concentration-dependent manner (**Figure 1F**), consistent with its Na channel-blocking effect. In hiPSC-CMs from the healthy donor, disopyramide showed similar effects on AP parameters (**Figure S1**).

**Figure 2:**
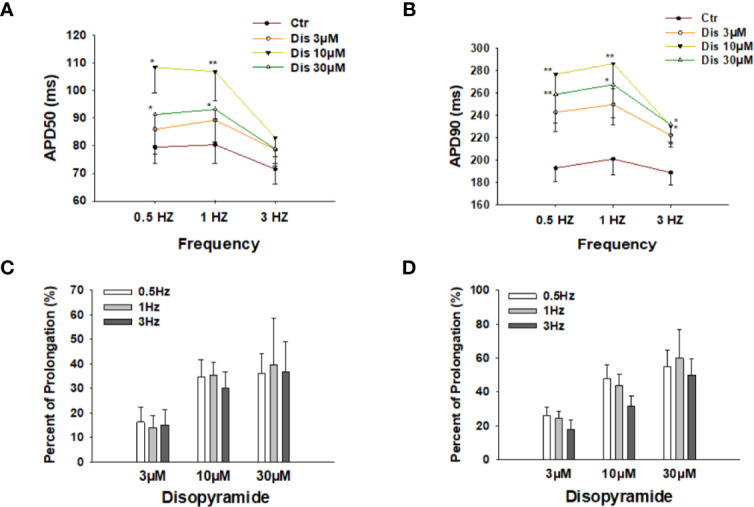
Effects of disopyramide on action potential duration (APD) at different frequencies in SQTS1-hiPSC-CMs. **(A, B)** Averaged values of APD50 and APD90 at 0.5 Hz, 1 Hz, and 3 Hz in absence (Ctr) and presence of 3 µM, 10 µM and 30 µM disopyramide. **(C, D)** Percent prolongation of APD50 and APD90 by disopyramide at 0.5 Hz, 1 Hz, and 3 Hz. The values were calculated from the data in **(A, B)**. Shown are mean ± SEM. The statistical significance was examined by One Way Repeated Measures ANOVA followed by Holm-Sidak method. *p<0.05, **p<0.01.

To check the effects of disopyramide at different beating frequencies, the same measurements were repeated in cells paced by stimulations at 0.5 Hz, 1 Hz, and 3 Hz. As expected, disopyramide prolonged APDs at all the three frequencies without clear frequency-dependence (**Figure 2**).

### Disopyramide Reduced Arrhythmic Events in SQTS1-hiPSC-CMs

**Figure 3:**
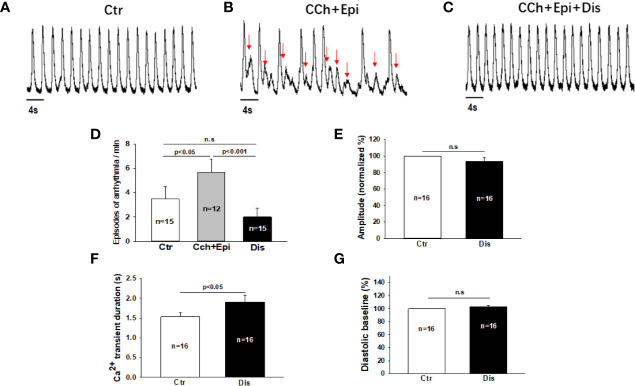
Disopyramide reduced arrhythmic events in SQTS1-hiPSC-CMs. Calcium transients were measured in spontaneously beating cells. Then carbachol (10 µM) plus epinephrine (10 µM) was applied to cells to trigger arrhythmic events. In cells showing arrhythmias, disopyramide (10 µM) was applied to the cell in presence of carbachol and epinephrine. **(A)** Representative traces of calcium transients in a cell before challenging (Ctr). **(B)** Representative traces of calcium transients in the cell challenged by carbachol plus epinephrine (CCh+Epi). **(C)** Representative traces of calcium transients in the cell in the presence of carbachol plus epinephrine and disopyramide (CCh+Epi+Dis). **(D)** Averaged values of arrhythmic events per minute. CCh+Epi slowed the beating but led to small and irregularly triggered beating. The arrhythmic events were defined as transients that are larger than 10% but smaller than 80% of the normal regular transients. The arrows indicate arrhythmic events. **(E)** Mean values of the amplitude of calcium transients in absence (Ctr) and presence of disopyramide (10 µM). **(F)** Mean values of the duration of calcium transients. **(G)** Mean values of the diastolic baseline of calcium transient. Shown are mean ± SEM, n represents number of cells. p values were determined by One Way ANOVA followed by Holm-Sidak method **(D)** or paired t-test **(E–G)**. ns, not significant.

Due to the APD-prolonging effect of disopyramide, its antiarrhythmic effects were further examined in SQTS1-hiPSC-CMs. In spontaneously beating cells challenged by carbachol (CCh, 10 µM) plus epinephrine (Epi, 10 µM), spontaneous calcium transients were measured to monitor arrhythmic events. CCh+Epi reduced the beating frequency but increased the episodes of arrhythmic events such as “immature” or irregularly triggered beats. Disopyramide reduced arrhythmic events induced by CCh+Epi (**Figures 3A–D**). Of note, the duration of calcium transients was prolonged but the amplitude and basal line of calcium transients were not changed (**Figures 3E–G**).

### Disopyramide Failed to Suppress the HERG Channel Current in SQTS1-hiPSC-CMs

**Figure 4:**
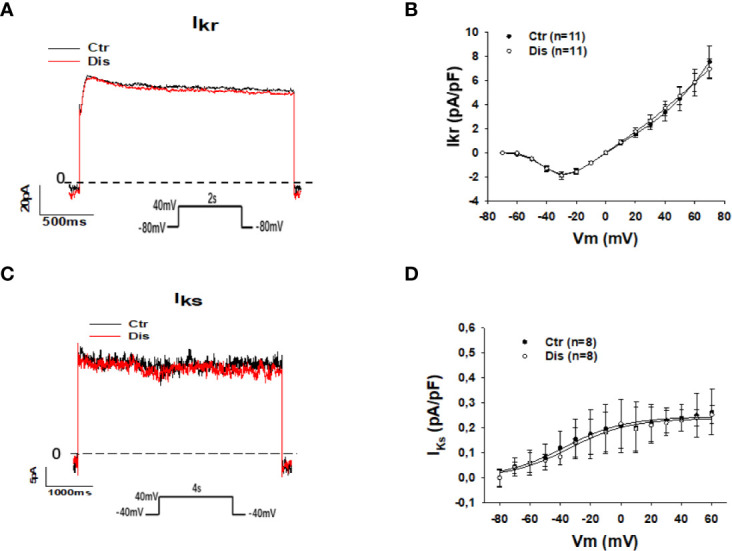
Effects of disopyramide on I_Kr_ and I_Ks_ in SQTS1-hiPSC-CMs. The currents (I_Kr_ and I_Ks_) were evoked by the protocol indicated in **(A, C)**. I_Kr_ was measured as Cs^+^ currents. I_Ks_ was analyzed as Chromalol-293B (10 µM) sensitive currents. **(A)** Representative traces of I_Kr_ in absence (Ctr) and presence of disopyramide (10 µM). **(B)** I-V curves of I_Kr_ in absence (Ctr) and presence of disopyramide. **(C)** Representative traces of I_Ks_ in absence (Ctr) and presence of disopyramide (10 µM). **(D)** I-V curves of I_Ks_ in absence (Ctr) and presence of disopyramide. n, number of cells.

The prolongation of action potential duration (APD) in SQTS1-hiPSC-CMs observed in this and previous study (Shinnawi et al., 2019) and the weak influence of the pathogenic mutation N588K in HERG channels on the channel-blocking by disopyramide (McPate et al., 2006) suggest that disopyramide should inhibit the HERG channel currents (I_Kr_). Surprisingly, disopyramide up to 30 µM failed to suppress I_Kr_ in SQT1-hiPSC-CMs under our conditions (**Figures 4A, B**). Likewise, disopyramide displayed no effects on the slowly activating delayed rectifier potassium channel current (*I_Ks_*) (**Figures 4C, D**). In hiPSC-CMs from the healthy donor, disopyramide inhibited significantly I_Kr_ (**Figure S2**).

### Disopyramide Reduced Small Ca^2+^-Activated K^+^ Currents in SQTS1-hiPSC-CMs

**Figure 5:**
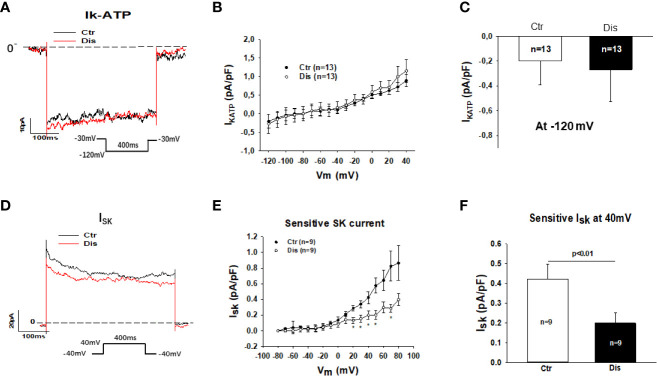
Effects of disopyramide on I_KATP_ and I_SK_ in SQTS1-hiPSC-CMs. The currents (I_KATP_ and I_SK_) were evoked by the protocol indicated in **(A, D)**. I_KATP_ was measured as nicorandil (10 µM) sensitive currents. I_SK_ was analyzed as apamin (100 nM) sensitive currents. **(A)** Representative traces of I_KATP_ in absence (Ctr) and presence of disopyramide (10 µM) at -120 mV. **(B)** I-V curves of I_KATP_ in absence (Ctr) and presence of disopyramide. **(C)** Mean values of I_KATP_ at -120 mV in absence (Ctr) and presence of disopyramide (10 µM). **(D)** Representative traces of I_SK_ at +40 mV in absence (Ctr) and presence of disopyramide (10 µM). **(E)** I-V curves of I_SK_ in absence (Ctr) and presence of disopyramide. **(F)** Mean values of I_SK_ at +40 mV in absence (Ctr) and presence of disopyramide (10 µM). n, number of cells. *p<0.05.

Then, we assessed other outward currents including the ATP-sensitive K channel current (I_KATP_) and the small conductance Ca^2+^-activated K^+^ channel current (I_SK_). Disopyramide at the highest concentration (30 µM) used in this study showed no significant effects on I_KATP_ but it reduced I_SK_ already at 10 µM (**Figure 5**). In hiPSC-CMs from the healthy donor, disopyramide inhibited also I_SK_ (**Figure S3**).

### Disopyramide Enhanced L-Type Calcium Channel Currents in SQTS1-hiPSC-CMs

**Figure 6:**
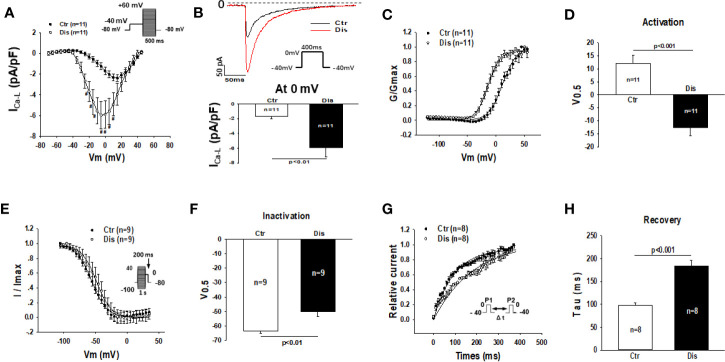
Effects of disopyramide on L-type calcium channel currents in SQTS1-hiPSC-CMs. The L-type Ca channel currents (I_Ca-L_) were evoked by the protocol indicated in **(A)**. **(A)** Current-voltage relationship (I-V) curves of I_Ca-L_ in absence (Ctr) and presence of disopyramide (10 µM). **(B)** The representative traces of I_Ca-L_ (upper panel) and current density (bottom panel) at 0 mV in absence (Ctr) and presence of disopyramide. **(C)** The activation curves of I_Ca-L_. **(D)** Mean values of the potential at 50% activation (V0.5). **(E)** The inactivation curves of I_Ca-L_. **(F)** Mean values of the potential at 50% inactivation (V0.5). **(G)** The curves of recovery of I_Ca-L_ from inactivation. **(H)** Mean values of the time constants (tau) of the recovery curves. Shown are mean ± SEM, n represents number of cells. ^#^p<0.05.

Disopyramide (10 µM) was applied to SQTS1-hiPSC-CMs through an extracellular perfusion-system to check the drug effects on L-type calcium channel currents (I_Ca-L_) evoked by stimulations at a fixed frequency (1 Hz). The I_Ca-L_ was significantly enhanced by 10 µM disopyramide (**Figures 6A, B**). The activation curve of I_Ca-L_ was largely shifted to more negative potentials and the inactivation curve was only slightly shifted to more positive potentials, whereas the recovery from inactivation was decelerated (**Figures 6C–H**). In hiPSC-CMs from the healthy donor, disopyramide inhibited significantly I_Ca-L_ (**Figure S4**).

### Disopyramide Enhanced the Na/Ca Exchanger Currents in SQTS1-hiPSC-CMs

**Figure 7:**
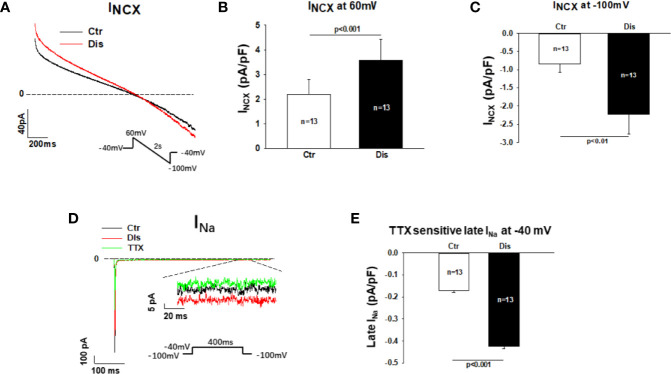
Effects disopyramide on late Na channel and Na/Ca exchanger currents in SQTS1-hiPSC-CMs. Late Na channel currents (late I_Na_) were evoked by the protocol indicated in **(D)** and measured at 300 ms after initiation of the depolarization pulse. TTX (30µM) sensitive currents were analyzed as late I_Na_. The Na/Ca exchanger currents (I_NCX_) were evoked by the protocol indicated in **(A)**. I_NCX_ was analyzed as NiCL2 (5mM) sensitive currents. **(A)** Representative traces of I_NCX_ in absence (Ctr) and presence of disopyramide (10 µM). **(B)** Mean values of I_NXC_ at +60 mV in absence (Ctr) and presence of disopyramide. **(C)** Mean values of I_NXC_ at -100 mV in absence (Ctr) and presence of disopyramide. **(D)** Representative traces of peak and late I_Na_ in absence (Ctr) and presence of disopyramide (10 µM). **(E)** Mean values of late I_Na_ at -40 mV in absence (Ctr) and presence of disopyramide. n, number of cells.

To separate the Na/Ca exchanger current (I_NCX_) from other currents, NiCl_2_ (5mM) was applied to cells and the NiCl_2_-sensitive currents were defined as I_NCX_. Disopyramide (10 µM) increased I_NCX_, especially the inward current at negative potentials (**Figures 7A–C**). In hiPSC-CMs from the healthy donor, disopyramide enhanced also I_NCX_ (**Figure S5**).

### Disopyramide Enhanced the Late Na Channel Currents in SQTS1-hiPSC-CMs

Measuring late Na channel current is challenging due to its small amplitude. To improve the measurements and reduced the influence of other currents, high concentration of extracellular Na concentration (140 mM) was used to increase the driving force and TTX (30µM)-sensitive currents (late I_Na_) were analyzed at 300 ms after initiation of depolarizing pulse. Under this condition 10 µM disopyramide enhanced significantly the late I_Na_, although it suppressed the peak I_Na_ (**Figures 7D, E**). In hiPSC-CMs from the healthy donor, disopyramide exerted similar effects (**Figure S6**).

## Discussion

In the current study, for the first time, we investigated the ionic mechanism underlying the APD-prolonging and antiarrhythmic effects of disopyramide in hiPSC-CMs from a patient with SQTS type 1. The new findings in this study are: (1) Disopyramide enhanced I_Ca-L_, I_NCX_ and late I_Na_ in SQTS1-hiPSC-CMs; (2) Disopyramide reduced I_SK_ in SQTS1-hiPSC-CMs. These effects may underlie the APD-prolonging and antiarrhythmic effect of disopyramide.

Disopyramide is a class Ia antiarrhythmic drug used in the therapy of atrial and ventricular arrhythmias. A major feature of class Ia antiarrhythmic drugs is a use-dependent block of the cardiac fast sodium current, which underlies the suppression of excitability and conduction speed (Sunami et al., 1991; Zunkler et al., 2000). The sodium channel blocking is an important effect of class I antiarrhythmic drugs including disopyramide for terminating tachyarrhythmias.

Disopyramide has been reported to prolong the QTc interval and is considered to be a promising agent for normalizing the QTc by blocking potassium channels (Dritsas et al., 1992; Sherrid et al., 2005; Schimpf et al., 2007; Johnson et al., 2011).

Disopyramide has also anticholinergic effect that may contribute to QT prolongation (Mirro et al., 1980; Teichman, 1985). At cellular level, disopyramide has been shown to prolong APD (Shinnawi et al., 2019), consistent with the QT-prolongation at organ level (Mizobuchi et al., 2008). The APD-and QT-prolongation could be another reason for disopyramide effect terminating arrhythmias.

In the current study, we used hiPSC-CMs from a patient with SQTS1 to examine the APD-prolonging and antiarrhythmic effects of disopyramide, mainly the ionic mechanisms of the effects. In the SQTS1-hiPSC-CMs, disopyramide changed significantly APs, mainly the Vmax, APD50, and APD90 and reduced arrhythmic events induced by CCh+Epi. The reduction of Vmax is consistent with its Na channel blocking effect (suppressing peak I_Na_). Both the reduction of Vmax and the APD-prolongation may contribute to the antiarrhythmic effect of disopyramide in SQTS1-hiPSC-CMs. However, how the APD was prolonged by disopyramide in SQTS1-hiPSC-CMs needs to be clarified.

I_Kr_ is an important repolarizing current for determining APD and is the target of many drugs that influence APD. Although the effect of disopyramide on I_Kr_ has already been examined in different types of cells (Virag et al., 1998; El Harchi et al., 2012b), whether the APD-prolongation in SQTS1-hiPSC-CMs resulted from the suppression of I_Kr_ by disopyramide is still unknown. The effect of disopyramide on I_Kr_ has not been tested in SQTS1-hiPSC-CMs. In the current study, we examined the effects on I_Kr_ in our SQTS1-hiPSC-CMs carrying the HERG channel mutation N588K, which has been well investigated and confirmed as a pathogenic mutation for SQTS1 (El-Battrawy et al., 2018; Shinnawi et al., 2019). The result is surprising, showing that disopyramide did not significantly reduce I_Kr_ in our SQTS1-hiPSC-CMs, although it suppressed I_Kr_ in donor-hiPSC-CMs. The HERG channel mutation (N588K) in our cells could be a reason for the ineffectiveness of disopyramide because the mutation has been shown to render I_Kr_ resistant to other drugs (McPate et al., 2008; Shinnawi et al., 2019). Of note, in CHO cells expressing I_Kr_ channels carrying N588K, disopyramide inhibited I_Kr_ with an efficacy similar to that in cells expressing wild type I_Kr_ channels (McPate et al., 2006), indicating the mutation did not change the efficacy of disopyramide in CHO cells. Considering the difference between CHO cells and cardiomyocytes, we expect that some unknown factors in addition to the gene mutations can influence the drug effects. Why disopyramide did not inhibit I_Kr_ in SQTS1-hiPSC-CMs remains to be clarified.

The failure of I_Kr_-blocking suggests extra ionic mechanisms for the APD-prolongation by disopyramide in SQTS1-hiPSC-CMs. Therefore, other ion channel currents that may influence APD were investigated.

Disopyramide was shown to block I_Kur_ (Arechiga et al., 2008), I_KAch_, and I_to_ (Virag et al., 1998), but those currents were found specifically or predominantly in atrial myocytes. It is unlikely that those three currents contribute to the APD-prolongation by disopyramide in ventricular-like hiPSC-CMs measured in this study. I_Ks_ and I_KATP_ can also influence APD in cardiomyocytes. Disopyramide did not change both currents in our study, indicating that both currents are not involved in the APD-prolongation by disopyramide. The I_KATP_, which is activated in ischemic/hypoxic conditions and leads to arrhythmogenic shortening of the APD, was reported to be suppressed by disopyramide (Horie et al., 1992; de Lorenzi et al., 1995). The disparity may result from differences of species or gene mutation related changes.

I_SK_, another outward K channel current, can also influence APD (Skibsbye et al., 2014; Zhang et al., 2014). We observed that disopyramide, indeed, reduced I_SK_ in our SQTS1- and donor-hiPSC-CMs, indicative of contribution of I_SK_ to the APD-prolongation in the presence of disopyramide.

Disopyramide was shown to inhibit I_Ca-L_ in rabbit sinus node cells and sheep Purkinje fibers (Coraboeuf et al., 1988; Kotake et al., 1988). The second surprising finding in this study is the enhancement of I_Ca-L_ by disopyramide in SQTS1-hiPSC-CMs. Since disopyramide inhibited I_Ca-L_ in donor-hiPSC-CMs, the reason for the disparity is probably the mutation or disease related alteration. To date, the effect of disopyramide on I_Ca-L_ has been not tested in cardiomyocytes from SQTS-patients. The enhancement of I_Ca-L_ in SQTS1-hiPSC-CMs by disopyramide, which may contribute to APD-prolongation, suggests that the mutation in KCNH2 may cause changes in channels in addition to the HERG channel.

The inward currents I_NCX_ and late I_Na_ may also influence APD. To our knowledge, disopyramide effect on I_NCX_ has not been reported. Inhibitory effect of disopyramide on late I_Na_ has been reported. A study showed that disopyramide inhibited persistent late human cardiac Na^+^ currents conducted by inactivation-deficient mutant Na^+^ channels (hNav1.5-CW mutant) expressed in HEK293 (Wang et al., 2013). Another study demonstrated that when the fast component of I_Na_ inactivation was removed by chloramine-T, I_Na_ amplitude was reduced by disopyramide (20 µM) (Koumi et al., 1992). Those data indicate that disopyramide can reduced late I_Na_. In current study, we observed that disopyramide enhanced I_NCX_ and late I_Na_ in both donor and SQTS1 hiPSC-CMs. How disopyramide enhanced both currents is not clear. Given that other types of sodium channels including SCN10A and SCN1B are also expressed in cardiomyocytes, it could be possible that disopyramide can activate SCN10A or other ion channels or proteins that can enhance late I_Na_. Although exact mechanisms for the surprising findings in this study need to be clarified in future studies, these effects may help explain the APD-prolongation by disopyramide.

Recently, a study (Blinova et al., 2019) examined effects of two drugs (dofetilide and moxifloxacin) on QT-intervals in subjects and action potential durations in hiPSC-CMs from the same subjects. The study showed no significant correlation between the subject-specific APD response slopes and clinical QT response slopes to either moxifloxacin (P = 0.75) or dofetilide (P = 0.69). Similarly, no significant correlation was found between baseline QT and baseline APD measurements (P = 0.93). These results facilitate discussion into factors obscuring correlation between hiPSC-CM studies and clinical studies. In addition, the study hinted at the possibility that immaturity and inherent variability of iPSC-CMs may dim patient-specific drug response prediction in the clinic. Hence, to know the real effects of disopyramide in SQTS1-patients, clinical studies are still necessary in spite of the results in SQTS1-hiPSC-CMs.

Other studies showed that disopyramide at 10 µM caused EAD-like events and at 100 µM caused ectopic beats (Bot et al., 2018) and possess a high TdP (Torsade de pointes) risk (Blinova et al., 2018). These studies suggest that when disopyramide, as many other antiarrhythmic drugs, is applied to patients, not only the antiarrhythmic but also the proarrhythmic effect should be considered.

In summary, in SQTS1-hiPSC-CMs, the ionic mechanisms of disopyramide behind APD-prolonging and antiarrhythmic effects were investigated. The results demonstrated that the APD-prolonging and antiarrhythmic effects of disopyramide in SQTS1-hiPSC-CMs with N588K-HERG channels resulted from enhancing I_Ca-L_, I_NCX_, late I_Na_, and reducing I_SK_ besides Na channel blocking.

### Study Limitations

Due to the difficulty to find SQTS1 patients from different families with the same mutation in HERG channels, we recruited only one SQTS1 patient for this study. Differences among individuals cannot be ruled out. However, the studies using cells from a single patient with a specific gene mutation may be also relevant, at least for precision medicine.

The immaturity is another limitation of hiPSC-CMs to be considered. Without studies in mature human cardiomyocytes, the possibility that disopyramide displays effects in native cardiomyocytes of SQTS-patients different from that of the SQTS1-hiPSC-CMs cannot be excluded.

Given the difference between hiPSC-CMs and adult human cardiomyocytes as well as the limitation of study in cells from only one patient, the clinical efficacy of disopyramide still needs to be examined in SQTS1-patients.

## Data Availability Statement

The raw data supporting the conclusions of this article will be made available by the authors, without undue reservation.

## Ethics Statement

The studies involving human participants were reviewed and approved by Ethics Committee of the Medical Faculty Mannheim, University of Heidelberg (Stated in the manuscript). The patients/participants provided their written informed consent to participate in this study.

## Author Contributions

HL, QX, XL, RZ, and SL performed experiments and analyzed data. IE-B, LC, MB, XZ, and IA designed the study and wrote the paper. All authors contributed to the article and approved the submitted version.

## Funding

This study was supported by the German Center for Cardiovascular Research (DZHK) (81Z0500204) and National Natural Science Foundation of China (No. 31300947).

## Conflict of Interest

The authors declare that the research was conducted in the absence of any commercial or financial relationships that could be construed as a potential conflict of interest.
