# Supplementary material for: Ionic Mechanisms of Disopyramide Prolonging Action Potential Duration in Human-Induced Pluripotent Stem Cell-Derived Cardiomyocytes From a Patient With Short QT Syndrome Type 1
Source: Front Pharmacol. 2020 Oct 12;11:554422. doi: 10.3389/fphar.2020.554422 (PMC7586889; doi:10.3389/fphar.2020.554422)
Supplement: Supplementary file 1 [file DataSheet_1.pdf]

Supplementary information

Ionic mechanisms of disopyramide prolonging action potential duration in human-induced pluripotent stem cell-derived cardiomyocytes from a patient with short QT syndrome type 1

Running title: Lan et al, disopyramide effects in SQTS1-hiPSC-CMs

Huan Lan[1*], Qiang Xu[2,5*], Ibrahim El-Battrawy[2,3], Rujia Zhong[2], Xin Li[2], Siegfried Lang[2,3], Lukas Cyganek[4,3], Martin Borggrefe[2,3], Xiaobo Zhou[2,3,1], Ibrahim Akin[2,3]

[1]Key Laboratory of Medical Electrophysiology of Ministry of Education and Medical Electrophysiological Key Laboratory of Sichuan Province, Institute of Cardiovascular Research, Southwest Medical University, Luzhou, China. [2]First Department of Medicine, Faculty of Medicine, University Medical Centre Mannheim (UMM), University of Heidelberg, Mannheim, Germany; [3]DZHK (German Center for Cardiovascular Research), Partner Sites, Heidelberg-Mannheim and Göttingen, Germany; [4]Stem Cell Unit, Clinic for Cardiology and Pneumology, University Medical Center Göttingen, Göttingen, Germany; [5]Department of Histology and Embryology, Southwest Medical University, Luzhou, China.

*equally contributed.

Address for correspondence:

Xiaobo Zhou, MD

First Department of Medicine, Faculty of Medicine, University Medical Centre Mannheim, University of Heidelberg, Theodor-Kutzer-Ufer 1-3, 68167 Mannheim, Germany. **E-mail**: Xiaobo.zhou@medma.uni-heidelberg.de

Funding:

This study was supported by the German Center for Cardiovascular Research (DZHK) (81Z0500204) and National Natural Science Foundation of China (No. 31300947).

Conflict of Interest: *The authors declare that the research was conducted in the absence of any commercial or financial relationships that could be construed as a potential conflict of interest*.

Supplemental Table 1. Comparison of effects of disopyramide in healthy and SQTS-cells

	Healthy cells	SQTS cells
APD	prolonged	prolonged
Vmax	reduced	reduced
Peak INa	reduced	reduced
Late INa	Reduced[1-2] , increased	increased
ICa-L	reduced	increased
Ito	Reduced[3]	NA
IKr	reduced	No effect
IKs	Reduced[4]	No effect
IK1	Increased[5]	NA
IKATP	Reduced[6-7]	No effect
ISK	No effect[8], reduced	reduced
INCX	increased	increased

NA: not analyzed

1. Wang et al. Persistent human cardiac Na^+ currents in stably transfected mammalian cells. Channels (Austin). Jul-Aug 2013;7(4):263-74. doi: 10.4161/chan.25056

2. S Koumi, R Sato, I Hisatome, H Hayakawa, H Okumura, R Katori. Disopyramide Block of Cardiac Sodium Current After Removal of the Fast Inactivation Process in Guinea Pig Ventricular Myocytes. J Pharmacol Exp Ther. 1992 Jun;261(3):1167-74.

3. Virag, L., Varro, A., and Papp, J.G., Effect of disopyramide on potassium currents in rabbit ventricular myocytes. Naunyn-Schmiedeberg's archives of pharmacology 357 (1998) 268-75.

4. Satoh H. Comparative Actions of Cibenzoline and Disopyramide on I(Kr) and I(Ks) Currents in Rat Sino-Atrial Nodal Cells. Eur J Pharmacol . 2000 Oct 27;407(1-2):123-9. doi: 10.1016/s0014-2999(00)00734-2.

5. Martin et al. Effects of Disopyramide and Flecainide on the Kinetics of Inward Rectifier Potassium Channels in Rabbit Heart Muscle. Br J Pharmacol 1994 Mar;111(3):873-9. doi: 10.1111/j.1476-5381.1994.tb14819.x.
6. Horie, M., Hayashi, S., Yuzuki, Y., and Sasayama, S., Comparative studies of ATP sensitive potassium channels in heart and pancreatic beta cells using Vaughan-Williams class Ia antiarrhythmics. Cardiovascular research 26 (1992) 1087-94.

7. de Lorenzi, F.G., Bridal, T.R., and Spinelli, W., Voltage-dependent inhibition of the ATP-sensitive K+ current by the class Ia agent disopyramide in cat ventricular myocytes. The Journal of pharmacology and experimental therapeutics 272 (1995) 714-23.
8. Rafel Simó-Vicens [1], Daniel R P Sauter [1], Morten Grunnet [2], Jonas G Diness [2], Bo H Bentzen. Effect of Antiarrhythmic Drugs on Small Conductance Calcium - Activated Potassium Channels. Eur J Pharmacol . 2017 May 15;803:118-123. doi: 10.1016/j.ejphar.2017.03.039. Epub 2017 Mar 18.

Figure legends

Figure S1. Effects of disopyramide on action potentials in hiPSC-CMs from a healthy donor. Action potentials were recorded at 1 Hz. Disopyramide (10 µM) was applied to cells. (A) Representative action potential traces in absence (Ctr) and presence of 10 µM disopyramide. (B) Averaged values of action potential duration at 50% repolarization (APD50). (C) Averaged values of action potential duration at 90% repolarization (APD90). (D) Averaged values of resting potential (RP). (E) Averaged values of action potential amplitude (APA). (F) Averaged values of maximal depolarization velocity (Vmax). Shown are mean ± SEM, n represents number of cells. The statistical significance was examined by paired t-test.

Figure S2. Effects of disopyramide on I_{Kr} hiPSC-CMs from the healthy donor. I_{Kr} was measured as Cs^+ currents. (A) Representative traces of I_{Kr} at +40 mV evoked by the indicated protocol (inset) in absence (Ctr) and presence of disopyramide (10 µM). (B) Mean values of IKr at +40 mV in absence (Ctr) and presence of disopyramide (10 µM). n, number of cells. The statistical significance was examined by paired t-test.

Figure S3. Effects of disopyramide on I_{SK} in hiPSC-CMs from the healthy donor. The currents (I_{SK}) were evoked by the protocol indicated in A (inset). I_{SK} was analyzed as apamin (100 nM) sensitive currents. (A) Representative traces of I_{SK} at +40 mV in absence (Ctr) and presence of disopyramide (10 µM). (B) I-V curves of I_{SK} in absence

(Ctr) and presence of disopyramide. (C) Mean values of I_{SK} at +40 mV in absence (Ctr) and presence of disopyramide (10 µM). n, number of cells. The statistical significance was examined by paired t-test.

Figure S4. Effects of disopyramide on L-type calcium channel currents in hiPSC-CMs from the healthy donor. The L-type Ca channel currents (I_{Ca-L}) were evoked by the protocol indicated in A. (A) The representative traces of I_{Ca-L} at 0 mV in absence (Ctr) and presence of disopyramide (10 µM). (B) Current-voltage relationship (I-V) curves of I_{Ca-L} in absence (Ctr) and presence of disopyramide (10 µM). (C) Mean values of I_{Ca-L} at 0 mV in absence (Ctr) and presence of disopyramide (10 µM). shown are mean ± SEM, n represents number of cells. The statistical significance was examined by paired t-test.

Figure S5. Effects disopyramide on Na/Ca exchanger currents in hiPSC-CMs from the healthy donor. The Na/Ca exchanger currents (I_{NCX}) were evoked by the protocol indicated in A (inset). I_{NCX} was analyzed as NiCL2 (5mM) sensitive currents. (A) Representative traces of I_{NCX} in absence (Ctr) and presence of disopyramide (10 µM). (B) Mean values of I_{NXC} at +60 mV in absence (Ctr) and presence of disopyramide. (C) Mean values of I_{NXC} at -100 mV in absence (Ctr) and presence of disopyramide. n, number of cells. The statistical significance was examined by paired t-test.

Figure S6. Effects disopyramide on peak and late Na channel currents in hiPSC-CMs from the healthy donor. Peak and late Na channel currents (I_{Na}) were evoked by the protocol indicated in A (inset). Late I_{Na} was measured at 300 ms after initiation of the depolarization pulse. TTX (30µM) sensitive currents were analyzed as late I_{Na}. (A) Representative traces of I_{Na} in absence (Ctr) and presence of disopyramide (10 µM). (B) I-V curves of peak I_{Na} in absence (Ctr) and presence of disopyramide (10 µM).

(C) Mean values of peal I_{Na} at -40 mV in absence (Ctr) and presence of disopyramide.

(D) Mean values of late I_{Na} at -40 mV in absence (Ctr) and presence of disopyramide.

n, number of cells. The statistical significance was examined by paired t-test.

Figures

Figure S1

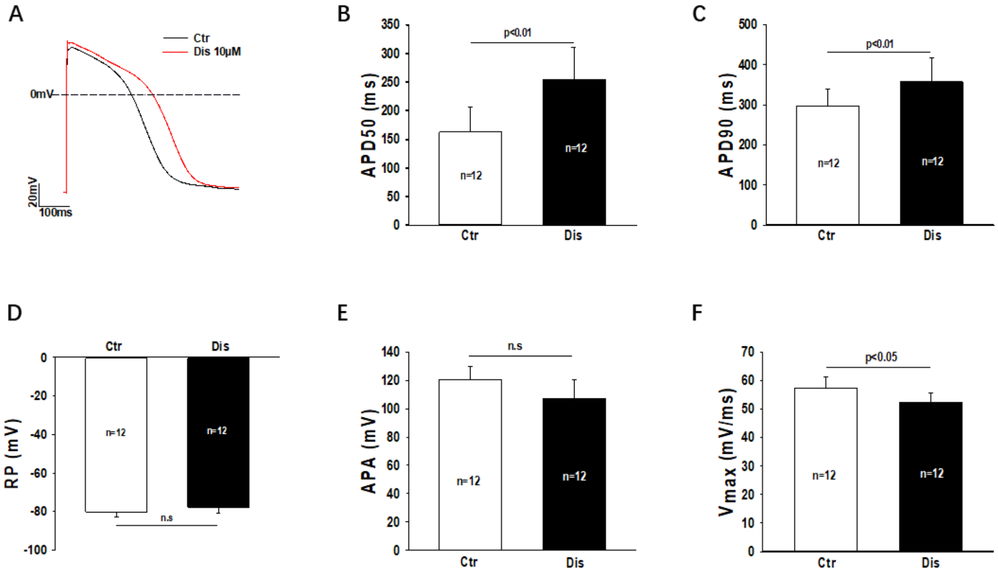

Figure S2

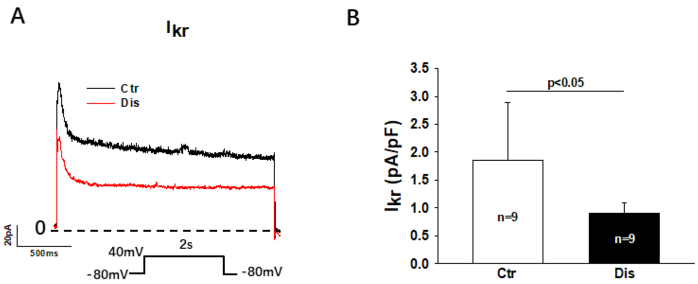
A
I_{kr}
Ctr
Dis
0
20pA
500ms
40mV
2s
-80mV
-80mV
B
3.5
3.0
2.5
2.0
1.5
1.0
0.5
0.0
I_{kr} (pA/pF)
p<0.05
n=9
n=9
Ctr
Dis

Figure S3

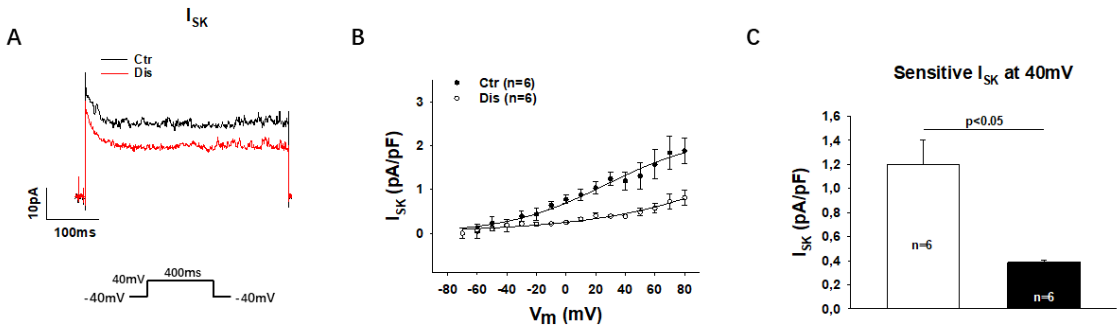

A
I_{SK}
Ctr
Dis
10pA
100ms
40mV
400ms
-40mV
-40mV
B
Ctr (n=6)
Dis (n=6)
I_{SK} (pA/pF)
3
2
1
0
-80 -60 -40 -20 0 20 40 60 80
V_m (mV)
C
Sensitive I_{SK} at 40mV
I_{SK} (pA/pF)
1,6
1,4
1,2
1,0
0,8
0,6
0,4
0,2
0,0
p<0.05
n=6
n=6

Figure S4

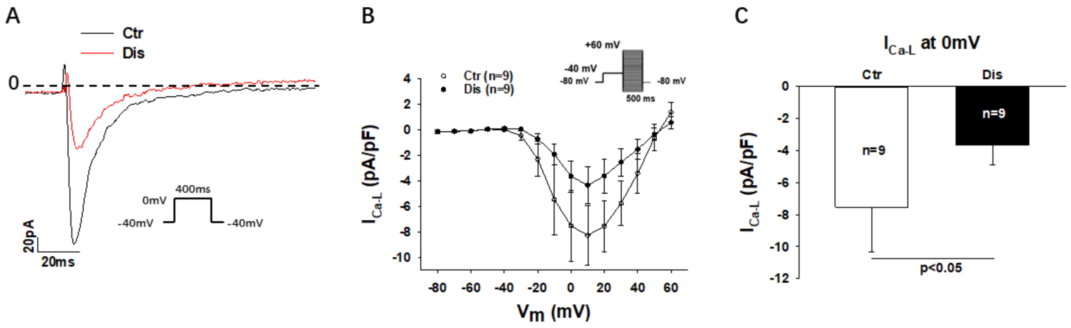
A
Ctr
Dis
0
400ms
0mV
-40mV
-40mV
20pA
20ms
B
+60 mV
-40 mV
-80 mV
-80 mV
500 ms
Ctr (n=9)
Dis (n=9)
I_{Ca-L} (pA/pF)
V_m (mV)
C
I_{Ca-L} at 0mV
Ctr
Dis
n=9
n=9
I_{Ca-L} (pA/pF)
p<0.05

Figure S5

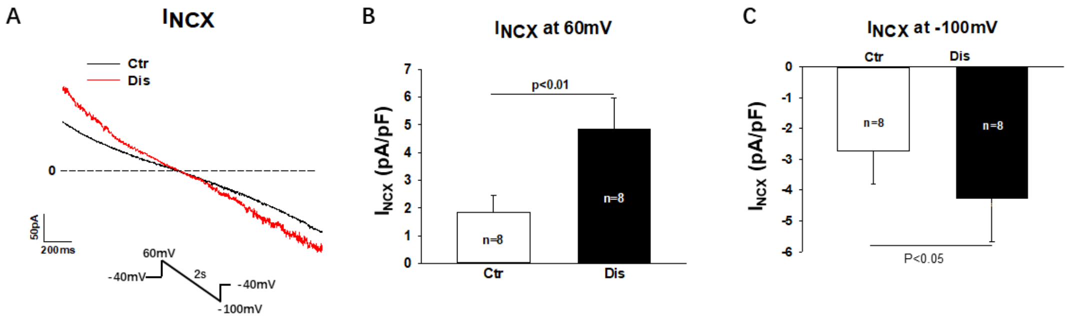
A
INCX
Ctr
Dis
0
50pA
200ms
60mV
-40mV
2s
-40mV
-100mV
B
INCX at 60mV
INCX (pA/pF)
p<0.01
n=8
n=8
Ctr
Dis
C
INCX at -100mV
Ctr
Dis
INCX (pA/pF)
n=8
n=8
P<0.05

Figure S6

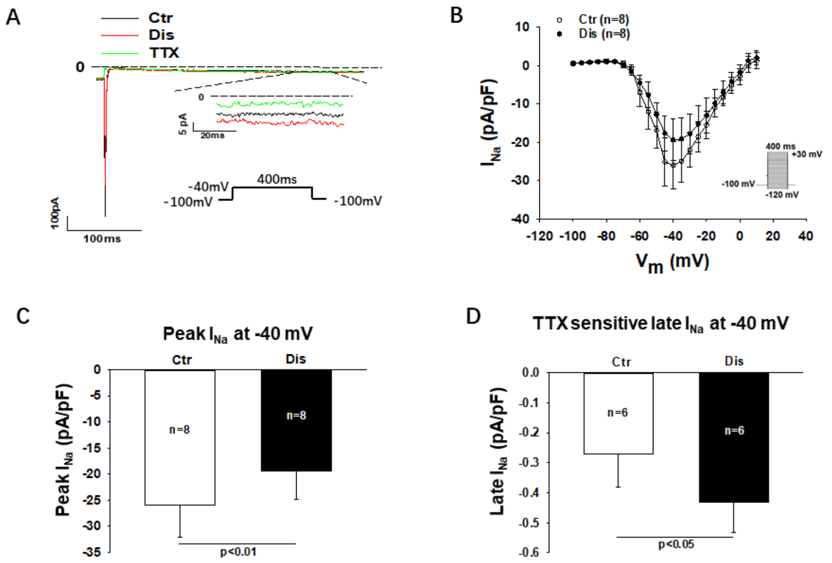

A
Ctr
Dis
TTX
0
5 pA
20ms
-40mV
400ms
-100mV
-100mV
100pA
100ms
B
Ctr (n=8)
Dis (n=8)
I_Na (pA/pF)
V_m (mV)
400 ms
+30 mV
-100 mV
-120 mV
C
Peak I_Na at -40 mV
Ctr
Dis
n=8
n=8
Peak I_Na (pA/pF)
p<0.01
D
TTX sensitive late I_Na at -40 mV
Ctr
Dis
n=6
n=6
Late I_Na (pA/pF)
p<0.05
